# Design, Utilization, and Impact of Smart Voice Interventions in Older Adults

**DOI:** 10.1093/geroni/igaa057.2218

**Published:** 2020-12-16

**Authors:** Megan Huisingh-Scheetz, Louise Hawkley, Shelia Cotten

## Abstract

Smart voice (voice-first) devices such as Amazon Alexa or Google Home devices use speech as the primary input method and employ artificial intelligence to “act” on spoken commands. Smart voice devices are thought to reduce technology use barriers for older adults because older users can utilize a skill they already have (talking) rather than learning a new skill (typing). Therefore, smart voice may be a promising technology vehicle for delivering social and functional resources and for assessing health in the home of older adults and their caregivers. However, very little clinical research has been conducted to understand the unique design considerations, utilization, potential impact, and limitations of smart voice applications in older adults. This symposium will 1) describe research methods and results of user-driven design of smart voice materials and programming in older adults; 2) quantify the utilization and potential impact of “EngAGE,” a smart voice-based program delivering socially-motivated exercise, on functional outcomes and summarize perceived benefits and challenges to use among older adult and caregiver participants following a feasibility study; and 3) detail key findings and policy implications following pilots of “Community Hub,” a data collection voice application to detect social isolation, and “Social Check-in,” programs leveraging smart voice to aid in older adult socialization.

